# Cyclic Dinucleotides in Oral Bacteria and in Oral Biofilms

**DOI:** 10.3389/fcimb.2017.00273

**Published:** 2017-06-21

**Authors:** Ulvi K. Gürsoy, Mervi Gürsoy, Eija Könönen, Herman O. Sintim

**Affiliations:** ^1^Department of Periodontology, Institute of Dentistry, University of TurkuTurku, Finland; ^2^Oral Health Care, Welfare DivisionCity of Turku, Turku, Finland; ^3^Department of Chemistry and Purdue Institute for Drug Discovery and Purdue Institute of Inflammation, Immunology and Infectious Disease, Purdue UniversityWest Lafayette, IN, United States

**Keywords:** caries, periodontitis, c-di-GMP, c-di-AMP, pathogens

## Abstract

Oral cavity acts as a reservoir of bacterial pathogens for systemic infections and several oral microorganisms have been linked to systemic diseases. Quorum sensing and cyclic dinucleotides, two “decision-making” signaling systems, communicate to regulate physiological process in bacteria. Discovery of cyclic dinucleotides has a long history, but the progress in our understanding of how cyclic dinucleotides regulate bacterial lifestyle is relatively new. Oral microorganisms form some of the most intricate biofilms, yet c-di-GMP, and c-di-AMP signaling have been rarely studied in oral biofilms. Recent studies demonstrated that, with the aid of bacterial messenger molecules and their analogs, it is possible to activate host innate and adaptive immune responses and epithelial integrity with a dose that is relevant to inhibit bacterial virulence mechanisms, such as fimbriae and exopolysaccharide production, biofilm formation, and host cell invasion. The aim of this perspective article is to present available information on cyclic dinucleotides in oral bacteria and in oral biofilms. Moreover, technologies that can be used to detect cyclic dinucleotides in oral biofilms are described. Finally, directions for future research are highlighted.

## Oral biofilms and cyclic dinucleotides

Oral biofilms form part of the healthy oral environment and there is a well-balanced equilibrium between bacteria and the host. Two most common infection-driven diseases of the oral cavity, periodontitis, and caries, are the outcomes of the disruption in this equilibrium. Periodontitis is a periodontal pathogen-induced disease, where chronic inflammation initiates degradation of tooth-supporting tissues, including the gingival epithelium, periodontal ligament, and alveolar bone (Darveau, 2010). Caries occurs when the acid metabolites of cariogenic bacteria dissolve dental structures: enamel and dentin. As a matter of fact, the oral cavity acts as a reservoir of bacterial pathogens for systemic infections and several oral microorganisms have been linked to systemic diseases, i.e., *Actinomyces* species to brain abscesses (Könönen and Wade, 2015), *Parvimonas micra* to septic arthritis (Baghban and Gupta, 2016), and *Streptococcus mutans* and *Porphyromonas gingivalis* to atherosclerotic plaques (Fernandes et al., 2014).

The oral cavity is a dynamic environment; eating, drinking, and even sleeping affect the pH, temperature, salivary flow, and nutrition. Depending on population dynamics and environmental factors, the relationship amongst bacteria in the biofilm could be symbiotic or competitive. Several studies have already indicated that oral bacteria can communicate with each other via quorum sensing molecules (Shao and Demuth, 2010; Sintim and Gürsoy, 2016). One important signaling mechanism for bacteria to sense environmental stress and respond adequately is secondary messenger molecules. In this context, cyclic dimeric guanosine 3′,5′-monophosphate (c-di-GMP), a bacterial secondary messenger molecule has become a molecule of high interest, since elevated concentrations of c-di-GMP regulate many processes affecting the initiation and maturation of bacterial biofilms (Valle et al., 2013). This occurs through the c-di-GMP-regulated production of exopolysaccharides, synthesis of extracellular adhesins, and fimbriae (Sondermann et al., 2012). C-di-GMP levels in bacteria are regulated through synthesis by diguanylate cyclases and degradation by phosphodiesterases (PDE). Diguanylate cyclases, which have a GGD/EEF domain (diguanylate cyclases can have GGDEF or GGEEF domain and GGD/EEF is a short hand for these two domains), make c-di-GMP via the condensation of two molecules of GTP. Degradation of c-di-GMP is regulated by PDEs, which contain EAL or HD-GYP domains. These metabolism proteins, together with c-di-GMP binding proteins, such as PilZ, modulate the intracellular perception of c-di-GMP (Ryjenkov et al., 2006). A recent complete genome analysis of oral biofilm-associated bacteria revealed that the genomes of some oral bacteria, including *Capnocytophaga ochracea, Prevotella melaninogenica, S. mutans, Streptococcus gordonii, Fusobacterium nucleatum, Aggregatibacter actinomycetemcomitans*, and *Filifactor alocis*, do not carry GGDEF or GGEEF, EAL, HDGYP, or PilZ domains, while some other oral bacteria, such as *Treponema denticola* and *Selenomonas sputigena*, do (Römling et al., 2013).

Recently, another bacterial second messenger, c-di-AMP has emerged as an important regulator of bacterial biofilm formation. This messenger molecule has a regulatory role in cell wall homeostasis, fatty acid synthesis, and biofilm formation (Opoku-Temeng et al., 2016). C-di-AMP is synthesized by diadenylate cyclases and degraded by PDEs. Several c-di-AMP receptors in various bacteria have been detected lately (Opoku-Temeng et al., 2016). However, there is a paucity of literature reports that have documented the presence of proteins containing diadenylate cyclase domains in oral bacteria.

Cyclic dinucleotide signaling in oral bacteria has been under-studied and, hence, there is almost no information about how cyclic dinucleotides affect oral bacterial physiology and virulence. This perspective highlights the dearth of cyclic dinucleotide research in this important class of bacteria.

### Cyclic dinucleotides in oral bacteria

#### Streptococcus mutans

*S. mutans*, a gram-positive facultatively anaerobic coccus, is one of the primary etiological agent of dental caries. Soluble and insoluble extracellular polysaccharides of *S. mutans* promote the formation and maturation of dental biofilms. These polysaccharides mediate the initial adherence of *S. mutans* to the hard surfaces and to other oral bacteria. *S. mutans* can convert dietary sugars into acids very efficiently. Inside biofilms, a constant accumulation of acids on tooth surface facilitates the demineralization of enamel (Selwitz et al., 2007). Colonization of *S. mutans* is not limited to the oral cavity. For example, it can act as an opportunistic pathogen and lead to infective endocarditis (Avilés-Reyes et al., 2017).

There is evidence that both c-di-GMP and c-di-AMP contribute to the regulation of oxidative response, extracellular polysaccharide matrix production, and biofilm formation of *S. mutans* (Yan et al., 2010; Cheng et al., 2016). Exogenous c-di-GMP was found to decrease adhesion and biofilm formation of *S. mutans* in a similar way as previously demonstrated for *Staphylococcus aureus* (Karaolis et al., 2005). According to the complete genome analyses of *S. mutans* UA159 (Ajdić et al., 2002), this bacterium does not have any GGD/EEF domain (Römling et al., 2013). However, Yan et al. (2010) reported that a conserved hypothetical protein, AAN59731 (encoded by the *gcp* gene), in *S. mutans* UA159 could act as a diguanyl cyclase when cloned into *E. coli*. The authors, however, did not provide any detailed data to substantiate this claim. AAN59731 does not contain the classical GGDEF domain so it appears that modified GGDEF domain proteins may also act as diguanyl cyclases. To conclusively demonstrate that AAN59731 is indeed a bona fide diguanyl cyclase, despite lack of a classical GGDEF domain, a biochemical and structural characterization of AAN59731, and detection of diguanyl cyclase activity and quantification of intracellular c-di-GMP when AAN59731 expression is induced are needed. In any case, a *gcp* knockout *S. mutans*, which is not able to produce AAN59731, does not form typical biofilms, providing further circumstantial evidence that AAN59731 could be a diguanyl cyclase (Yan et al., 2010).

Deletion of the *cdaA* gene coding for a diadenylate cyclase in *S. mutans* resulted in reduced c-di-AMP levels, an increased production of extracellular polysaccharides, and elevated sensitivity to hydrogen peroxide in this bacterium (Cheng et al., 2016). Yet, a confirmatory study on the role of the *cdaA* gene showed opposite results; the *cdaA*-mutant *S. mutans* produced less extracellular polysaccharides than the wild-type, thus questioning the relation between diadenylate cyclase and the extracellular polysaccharide production (Peng et al., 2016a). PDE that degrades c-di-AMP has been found in PdeA protein of *S. mutans*. Deletion of the *pdeA* gene (the gene that encodes c-di-AMP degrading PDE) or overexpression of diadenyl cyclase (the enzyme that produces c-di-AMP) increases intracellular c-di-AMP levels and biofilm formation (Peng et al., 2016b). C-di-AMP regulated extracellular polysaccharide production and biofilm formation are dependent on glucosyltransferases (Peng et al., 2016b).

#### Treponema denticola

*T. denticola* is an oral spirochete that is strongly associated with periodontitis. The *T. denticola* genome encodes proteins with GGD/EEF, EAL, HD-GYP, PilZ, and putative c-di-GMP binding proteins (Römling et al., 2013) and has at least two c-di-GMP binding proteins (Bian et al., 2013). Bioinformatic analyses reveal that each c-di-GMP signaling-related protein harbors diverse sensor domains, and these proteins according to biochemical analyses, enable the bacterium to sense and adopt environmental changes (Bian et al., 2013). Using a high performance liquid chromatography as the detection method, Kostick et al. (2011) reported the presence of intracellular c-di-GMP levels, while Bian et al. (2013) failed to detect c-di-GMP in *T. denticola*. The latter report suggested that TDE0214, a putative c-di-GMP binding protein in *T. denticola*, TDE0214, could regulate motility, chemotaxis, and biofilm-related virulence mechanisms of the bacterium. Moreover, lack of TDE0214 led to a reduced bacterial invasiveness and abscess formation of *T. denticola* in an *in vivo* mouse-infection model (Bian et al., 2013).

#### Porphyromonas gingivalis

*P. gingivalis* is an obligately anaerobic and mainly asaccharolytic bacterium. Even as a small part of periodontitis-associated biofilms, its ability to cause dysbiosis makes it a keystone pathogen in human periodontitis (Olsen and Hajishengallis, 2016). Although it has been previously predicted that there are no diguanylate cyclases in *P. gingivalis* (Römling et al., 2013), a more recent work has shown that *P. gingivalis* actually makes c-di-GMP (Chaudhuri et al., 2014). Some of the bacteria that have been predicted to lack a functional c-di-GMP system can indeed have one. Cyclic dinucleotide signaling in oral pathogens is worthy of further studies, since insights could lead to the development of novel therapies against oral biofilm-related infections.

## Methods to detect cyclic dinucleotides

Many methods have been developed to detect c-di-GMP in both cell lysates and in live bacteria. A high performance liquid chromatography and an ultra-performance liquid chromatography-tandem mass spectrometer are the methods of choice for a sensitive detection of c-di-AMP and c-di-GMP in bacterial cell lysates, but they both are inappropriate for live imaging of cyclic dinucleotides. A competitive enzyme-linked immunosorbent assay was developed as a simple method for the quantification of c-di-AMP levels in bacteria, however, the minimum detection limit (10 nm) of this method does not allow to measure c-di-AMP in culture media or in host cells (Underwood et al., 2014). Sintim and his co-workers demonstrated that low levels of c-di-GMP can be detected using a c-di-GMP riboswitch that was fused to a spinach aptamer (Nakayama et al., 2012). Later, the Hammond group adopted this design and modified a version of the riboswitch-spinach aptamer to detect c-di-GMP in live bacteria (Kellenberger et al., 2013). A limitation of this approach is the need to transform bacteria before c-di-GMP can be imaged.

Detection of intracellular cyclic dinucleotides in oral bacteria would allow us to understand their adaptive properties against a changing environment. However, oral bacteria live in multispecies biofilms where they have a continuous interaction, which potentially regulates their secondary messenger molecule formation and degradation. We tested this hypothesis in a dual bacterial co-culture model using *F. nucleatum* and four different selenomonads (*Selenomonas noxia, S. flueggei, S. artemidis*, and *S. sputigena*). *F. nucleatum* is a key species in dental biofilms by facilitating the colonization of periodontitis-associated pathogens by coaggregation-mediated mechanisms, and many *Selenomonas* spp. are known to coaggregate well with *F. nucleatum* (Kolenbrander et al., 1989). In our experiments, the four *Selenomonas* spp., i.e., *S. flueggei* (64 × 10^7^ CFU), *S. sputigena* (59 × 10^7^ CFU), *S. noxia* (64 × 10^7^ CFU), and *S. artemidis* (65 × 10^7^ CFU), were cultured, either alone or together with *F. nucleatum* (64 × 10^7^ CFU), in a reinforced clostridial medium at anaerobic conditions for 24 h. The bacterial cells were then centrifuged and the pellet was resuspended in 10 nm Tris-HCl containing 100 mM NaCl. Lysozyme (L6876, Sigma-Aldrich, St. Louis, USA) was added with a final concentration of 62.5 μg/ml. The samples were resuspended, sonicated, and heated to 90°C for 30 min, centrifuged once again, and extracted with CH_3_OH:CH_3_CN:H_2_O. The supernatants were centrifuged with Ultracel 3K centrifuge tubes (Amicon® Ultra-4 Centrifugal Filter Units, Merck Millipore, Germany) for 40 min at 4,000 g at a room temperature, and evaporated to dryness. Nucleotides were extracted and c-di-GMP levels were measured with ultra-performance liquid chromatography—tandem mass spectrometer.

**Figure 1:**
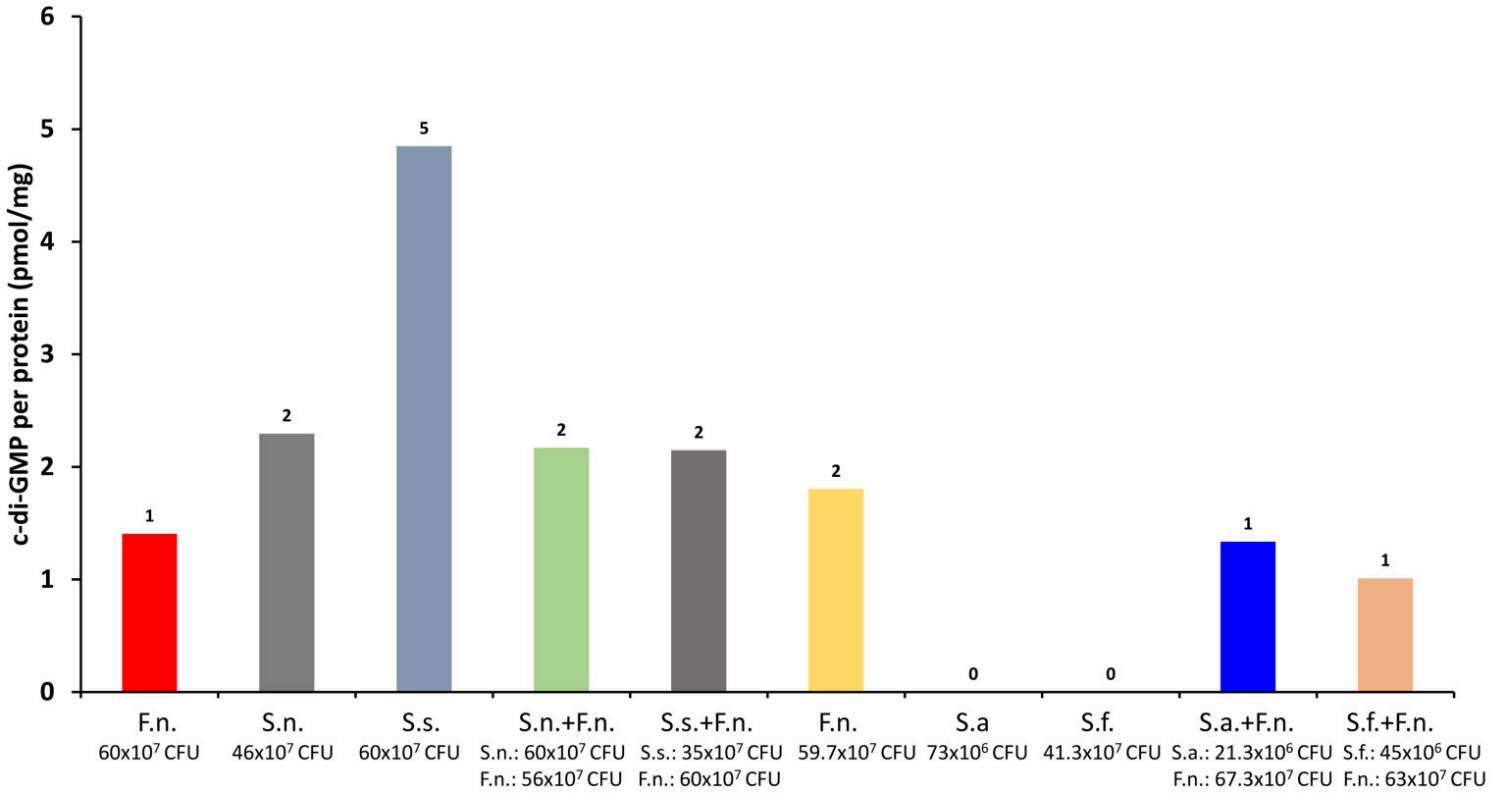
Low but detectable levels of c-di-GMP were detected. Whether the c-di-GMP were produced via bona fide synthases or from uncatalyzed condensations of GTP into c-di-GMP remain to be elucidated [*Fusobacterium nucleatum* (F.n.), *Selenomonas noxia* (S.n.), *Selenomosan sputigena* (S.s.), *S. artemidis* (S.a.), *S. flueggei* (S.f.)].

The numbers of bacterial cells in each group at the end of the experiment are given in Figure 1. According to our results, *S. sputigena* produced highest levels of c-di-GMP. Surprisingly, when co-cultured with *F. nucleatum*, c-di-GMP levels decreased nearly with 50%. This was an interesting finding, since it was expected that the co-culture of *S. sputigena* with *F. nucleatum* will increase its coaggregation and, hence, biofilm formation. However, reduced c-di-GMP levels indicate an increased motility and decreased biofilm formation (Figure 1).

To date, the reported technologies for detecting of c-di-GMP in live bacteria have not been powerful or simple enough to use for the simultaneous detection of c-di-GMP in polymicrobial biofilms. The ultra-performance liquid chromatography-tandem mass spectrometer is a post sampling method and the spinach-riboswitch method requires transfection, which may not work with all bacteria. Secondly, it is unclear whether the biology that emerges from a transfected bacterium represents what happens in a native scenario. The development of easy-to-use and broadly applicable c-di-GMP and c-di-AMP detection methods for various oral bacteria enables the delineation of how cyclic dinucleotides affect signaling in oral biofilms.

## Biofilm inhibition and cyclic dinucleotides

Disruption of biofilms by using molecules that interfere with cyclic dinucleotide regulation is a promising approach to fight against infectious diseases. In general, enhanced c-di-GMP levels intracellularly lead to biofilm formation, so it is expected that activators of PDE, inhibitors of diguanyl cyclases, or the supramolecular aggregation of the signaling molecules would inhibit formation (Kelsey et al., 2012; Nakayama et al., 2016). In this context, exogenous PDE was demonstrated to disperse *Pseudomonas aeruginosa* biofilms and reduce its biofilm formation (Hickman et al., 2005). Interestingly, the inhibition of RocR PDE in *P. aeruginosa* did not lead to enhanced biofilm formation but rather swarming inhibition, indicating that there are nuances to cyclic dinucleotide signaling and the interception of signaling with small molecules (Zheng et al., 2016). A few inhibitors of diguanylate cyclases have been identified or developed, and these were found to reduce bacterial biofilms (Opoku-Temeng et al., 2016). For example, diguanyl cyclase inhibitors can reduce *Vibrio cholerae* or *P. aeruginosa* biofilm formation (Sambanthamoorthy et al., 2012, 2014).

C-di-AMP is involved in biofilm formation as well as cell wall synthesis in gram-positive bacteria (Opoku-Temeng et al., 2016). The Sintim group has identified several small molecules that inhibit c-di-AMP synthases (Zheng et al., 2014; Opoku-Temeng and Sintim, 2016a,b) and some of these compounds inhibit the growth or biofilm formation of *S. aureus* (Opoku-Temeng et al., 2017).

## Conclusion

Cyclic dinucleotides were discovered already 30 years ago. During the past decade, a renewed interest in these fascinating second messengers led to a tremendous progress in our understanding of how cyclic dinucleotides regulate bacterial lifestyle but also bacteria-host interactions. Oral microorganisms form some of the most intricate biofilms, yet c-di-GMP and c-di-AMP signaling, which regulate biofilm formation, have been rarely studied in oral bacteria. Perhaps this dearth of information on dinucleotide signaling in oral bacteria is due to initial bioinformatics predictions, stating that many oral bacteria do not harbor proteins that are capable of making or degrading cyclic dinucleotides. Recent, but still few reports, however, challenge this perception, and c-di-GMP or c-di-AMP signaling has now been recognized from some oral bacterial species. We encourage the scientific community to take a second look at cyclic dinucleotide signaling in putative oral pathogens, as there might be potential drug targets in dinucleotides that could be usable in preventing oral biofilm-related diseases like periodontitis.

## Author contributions

UG, MG, EK, and HS: Substantial contributions to the conception or design of the work, drafting and critical revision of the article, and final approval of the version to be published.

### Conflict of interest statement

The authors declare that the research was conducted in the absence of any commercial or financial relationships that could be construed as a potential conflict of interest.
